# Structure of entinostat Form B, C_21_H_20_N_4_O_3_, derived using laboratory powder diffraction data and density functional techniques

**DOI:** 10.1107/S2056989025007406

**Published:** 2025-08-21

**Authors:** James A. Kaduk, Sunil Kumar Rai

**Affiliations:** a Department of Chemistry North Central College, 131 S Loomis St Naperville IL 60540 USA; b Department of Chemistry Faculty of Science University of Lucknow,Lucknow 226007 Uttar Pradesh India; University of Aberdeen, United Kingdom

**Keywords:** powder diffraction, entinostat, Rietveld refinement, density functional theory

## Abstract

The crystal structure of entinostat Form B, C_21_H_20_N_4_O_3_, has been solved and refined using laboratory X-ray powder diffraction data, and optimized using density functional techniques. Entinostat crystallizes in space group *Pna*2_1_ and the crystal structure consists of inter­locking layers of entinostat mol­ecules parallel to the *bc* plane. A strong N—H⋯N hydrogen bond links the mol­ecules into zigzag chains propagating along the *b*-axis direction. The graph set for this pattern is *C*^1^_1_(8). Two N—H⋯O hydrogen bonds link the mol­ecules along the *c*-axis direction. The graph sets for this pattern are *C*^1^_1_(4) and *C*^1^_1_(7).

## Abstract 2

The crystal structure of entinostat Form B has been solved and refined using laboratory X-ray powder diffraction data, and optimized using density functional techniques.

## 1. Chemical context

Entinostat, C_21_H_20_N_4_O_3_ (also known as SNDX-275 and MS-275), is undergoing clinic trials for treatment of various cancers. The rights to entinostat are owned by Syndax Pharmaceuticals. The systematic name (CAS Registry No. 209783-80-2) is pyridin-3-ylmethyl *N*-({4-[(2-amino­phen­yl)carbamo­yl]phen­yl}meth­yl)carbamate.
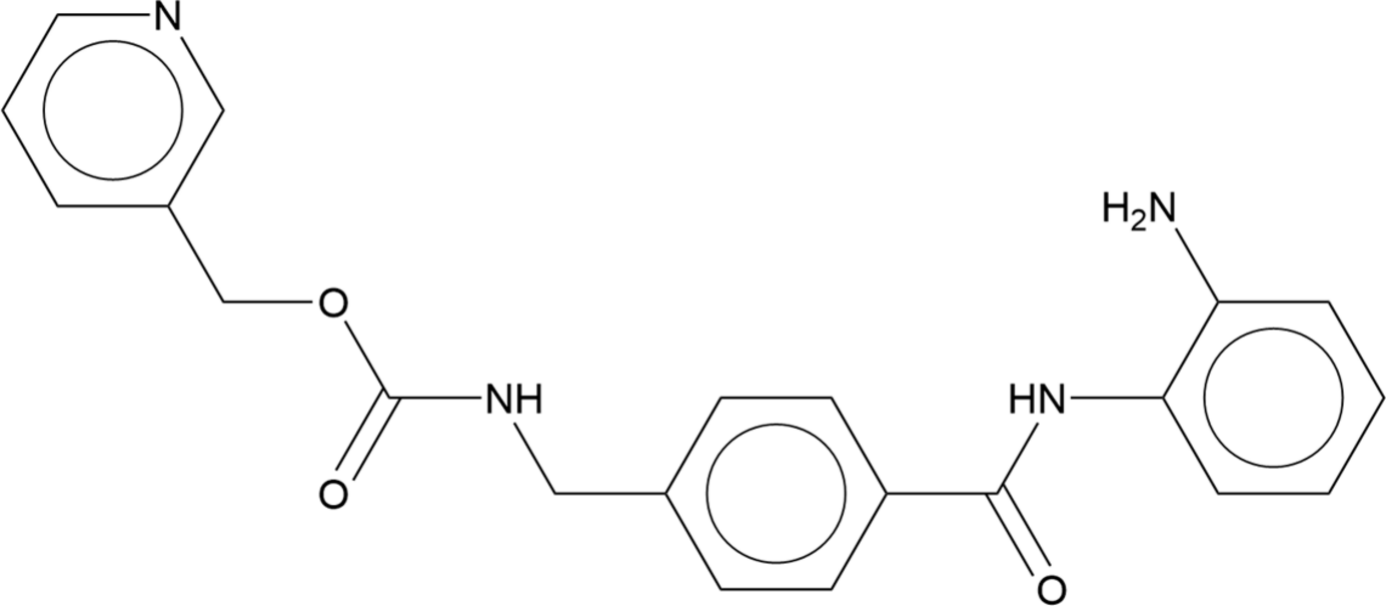


**Figure 1:**
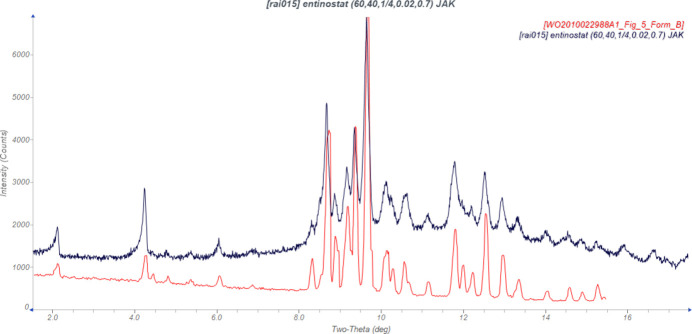
Comparison of the laboratory pattern of entinostat (measured using Mo *K*_α_ radiation; black) to that of Form B reported by Schneider *et al.* (2010 ▸; red). The patent pattern (measured using Cu *K*_α_ radiation) was digitized using *UN-SCAN-IT* (Silk Scientific, 2013 ▸) and converted to the Mo *K*_α_ wavelength of 0.7093187 Å using *JADE Pro* (MDI, 2025 ▸). Image generated using *JADE Pro* (MDI, 2025 ▸).

Inter­national Patent Application WO2010/022988 A1 (Schneider *et al.*, 2010 ▸; Bayer Schering Pharma) discloses crystalline Forms A, B, and C of entinostat and processes for their preparation. The material characterized here appears to be Form B (Fig. 1 ▸). Crystalline Forms D and E are disclosed in Inter­national Patent Application 2017/081278 A1 (Stefinovic & Reece, 2017 ▸; Sandoz AG). Stable amorphous entinostat is claimed in Inter­national Patent Application WO2017/216761 (Peddireddy *et al.*, 2017 ▸; Dr. Reddys Laboratories). Cocrystals of entinostat with maleic acid (Form A) and succinic acid (Forms A, B, and C) are claimed in US Patent Application US2024/023948 A1 (Bonnaud & Prentice, 2024 ▸; Macfarlan Smith Ltd.). This work was carried out as part of a project aimed at preparing cocrystals of active pharmaceutical ingredients using mechanochemical techniques.

## 2. Structural commentary

**Figure 2:**
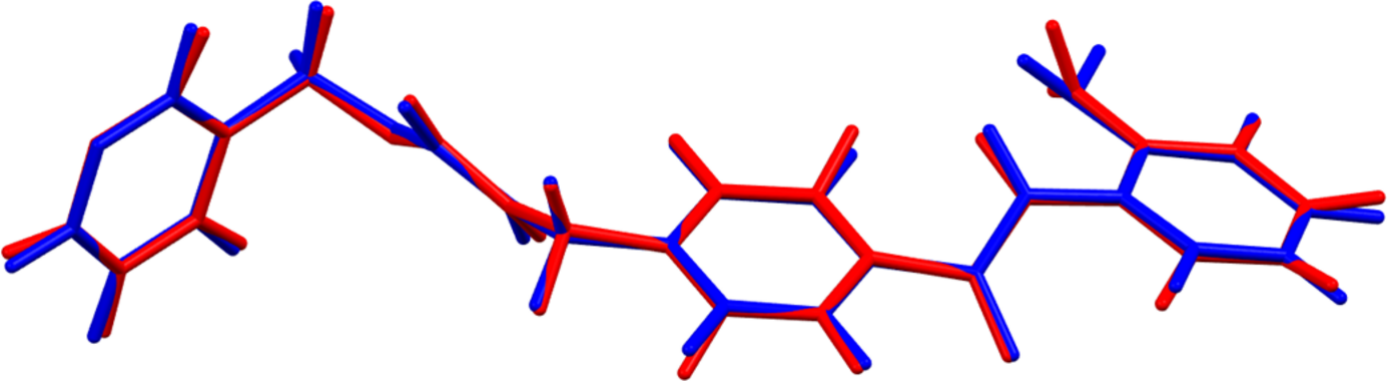
Comparison of the refined structure of the entinostat mol­ecule (red) to the VASP-optimized structure (blue). The comparison was generated using the *Mercury* calculate/mol­ecule overlay tool; the r.m.s. difference is 0.068 Å. Image generated using *Mercury* (Macrae *et al.*, 2020 ▸).

**Figure 3:**
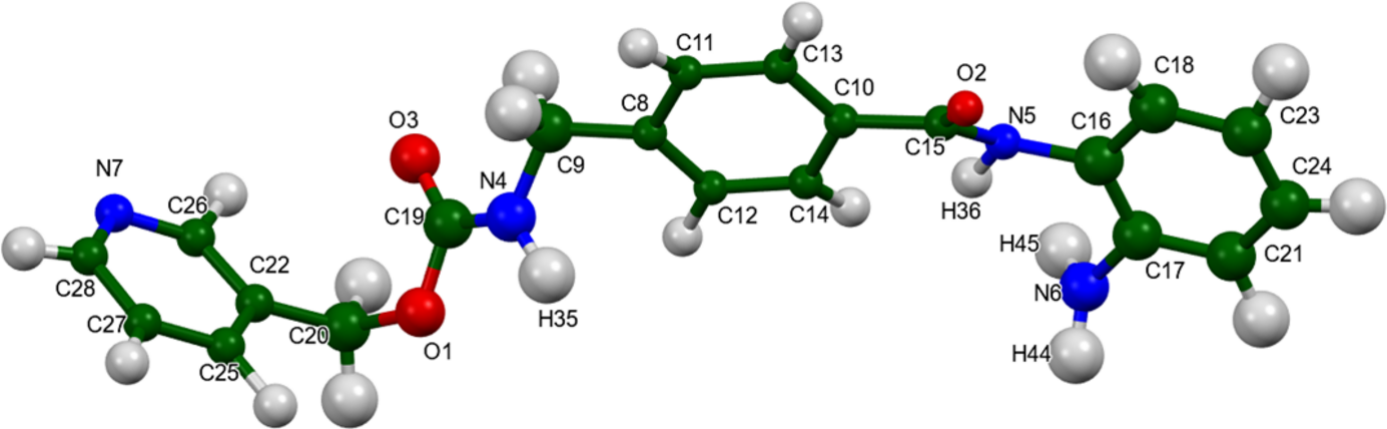
The asymmetric unit of entinostat Form B, with the atom numbering. The atoms are represented by 50% probability spheroids. Image generated using *Mercury* (Macrae *et al.*, 2020 ▸).

The root-mean-square Cartesian displacement between the Rietveld-refined and VASP-optimized structures is 0.068 Å (Fig. 2 ▸). The agreement is within the normal range for correct structures (van de Streek & Neumann, 2014 ▸). The asymmetric unit of the structure is illustrated in Fig. 3 ▸. The remaining discussion will emphasize the VASP-optimized structure.

All of the bond distances, bond angles, and torsion angles fall within the normal ranges indicated by a *Mercury* Mogul geometry check (Macrae *et al.*, 2020 ▸). Quantum chemical geometry optimization of the isolated entinostat mol­ecule (DFT/B3LYP/6-31G*/water) using *Spartan 24* (Wavefunction, 2023 ▸) indicated that the observed conformation is only 3.8 kcal mol^−1^ higher in energy than a local minimum, even though the r.m.s. displacement is 0.498 Å. The global minimum-energy conformation is much more compact (folded on itself to yield parallel phenyl rings), indicating that inter­molecular inter­actions are important in determining the solid-state conformation. The false minimum structures contained different conformations of the mol­ecule in roughly the same positions and orientation.

## 3. Supra­molecular features

**Figure 4:**
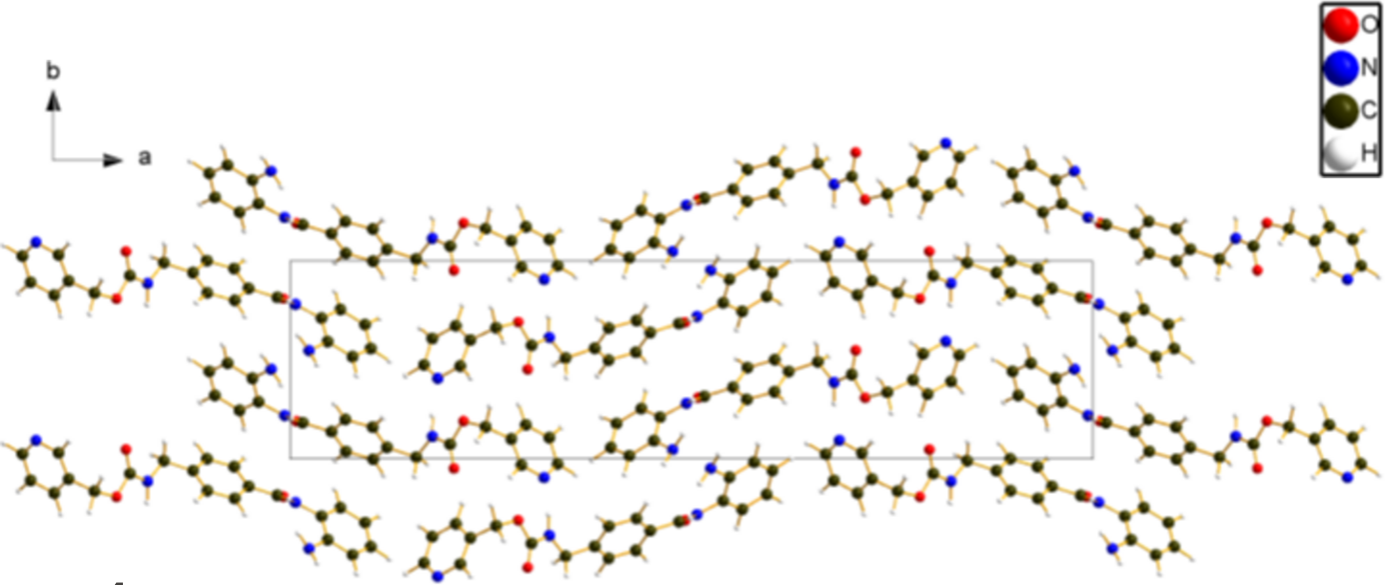
The crystal structure of entinostat Form B, viewed down the *c*-axis. Image generated using *DIAMOND* (Brandenburg & Putz, 2023 ▸).

The extended structure (Fig. 4 ▸) consists of inter­locking layers of entinostat mol­ecules lying parallel to the *bc* plane. Hydrogen bonds (discussed below) link the mol­ecules along the *b*- and *c*-axis directions. The mean planes of the amino­phenyl, phenyl, and pyridine rings correspond approximately to the (17,–5,–2), (13,–7,–3), and (26,–3,4) Miller planes, respectively. The *Mercury* aromatics analyser indicates strong inter­actions (centroid–centroid distance = 5.07 Å) between the two types of phenyl rings, moderate inter­actions with distances of 5.63 and 5.77 Å, as well as weaker inter­actions.

Analysis of the contributions to the total crystal energy of the structure using the Forcite module of *Materials Studio* (Dassault Systèmes, 2023 ▸) indicates that bond, angle, and torsion distortion terms contribute about equally to the intra­molecular energy. The inter­molecular energy is dominated by electrostatic attractions, which in this force field based analysis also include hydrogen bonds. The hydrogen bonds are better discussed using the results of the DFT calculation.

A strong N—H⋯N hydrogen bond links the mol­ecules into zigzag chains along the *b*-axis direction (Table 1 ▸). The graph set (Etter, 1990 ▸; Bernstein *et al.*, 1995 ▸; Motherwell *et al.*, 2000 ▸) for this pattern is 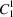
(8). Two N—H⋯O hydrogen bonds link the mol­ecules along the *c*-axis direction. The graph sets for this pattern are 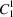
(4) and 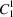
(7). The energies of the N—H⋯O hydrogen bonds were calculated using the correlation of Wheatley & Kaduk (2019 ▸). Several inter- and intra­molecular C—H⋯O hydrogen bonds, as well as one C—H⋯N hydrogen bond, contribute to the lattice energy.

**Figure 5:**
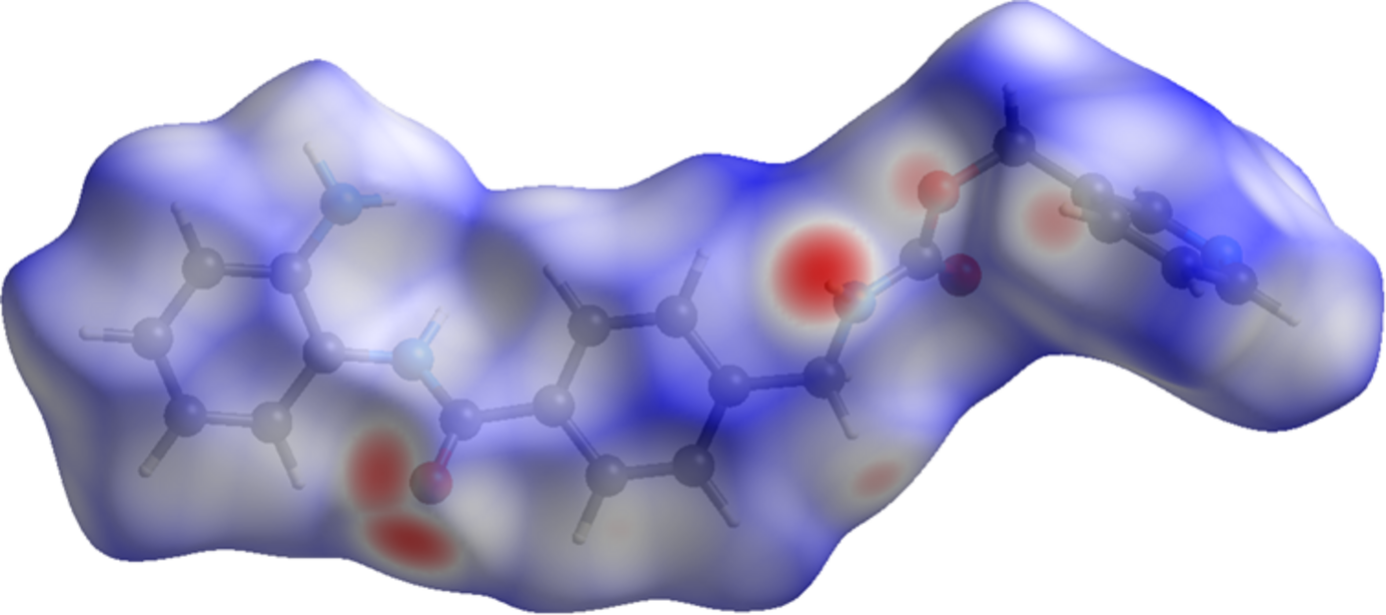
The Hirshfeld surface of entinostat Form B. Inter­molecular contacts longer than the sums of the van der Waals radii are colored blue, and contacts shorter than the sums of the radii are colored red. Contacts equal to the sums of radii are white. Image generated using *CrystalExplorer* (Spackman *et al.*, 2021 ▸).

The volume enclosed by the Hirshfeld surface of entinostat (Fig. 5 ▸, Hirshfeld, 1977 ▸, Spackman *et al.*, 2021 ▸) is 448.9 Å^3^ or 98.1% of 1/4 of the unit-cell volume. The packing density is thus fairly typical. The only significant close contacts (red in Fig. 5 ▸) involve the hydrogen bonds. The volume per non-hydrogen atom is smaller than normal, at 16.3 Å^3^.

The Bravais–Friedel–Donnay–Harker (Bravais, 1866 ▸; Friedel, 1907 ▸; Donnay & Harker, 1937 ▸) algorithm suggests that we might expect platy morphology for entinostat, with {200} as the principal faces. A second order spherical harmonic model was included in the refinement. The texture index was 1.002 (1), indicating that preferred orientation was not significant in the rotated capillary specimen.

## 4. Database survey

A reduced cell search in the Cambridge Structural Database (CSD, version 2025.1.0 May 2025; Groom *et al.*, 2016 ▸) yielded 33 hits, but no structures for entinostat or its derivatives. A connectivity search of the entinostat mol­ecule in the CSD yielded no hits. A search of the pattern against the Powder Diffraction File (Kabekkodu *et al.*, 2024 ▸) yielded no hits, and a name search on ‘entinostat’ also yielded no hits.

## 5. Synthesis and crystallization

The sample characterized here was obtained from a commercial source, was gently ground in a mortar and pestle and sieved to < 325 mesh.

## 6. Refinement

Crystal data, data collection and structure refinement details are summarized in Table 2 ▸. The pattern was indexed using *DICVOL14* (Louër & Boultif, 2014 ▸) on a primitive ortho­rhom­bic unit cell with *a* = 38.2913, *b* = 9.4545, *c* = 5.0779 Å, *V* = 1838.31 Å^3^, and *Z* = 4.

The space group was ambiguous. Space groups *Pna*2_1_, *Pca*2_1_, *Pba*2, and *P*2_1_2_1_2_1_ yielded similar profile fits, so the structure was solved in all of them using Monte Carlo-simulated annealing techniques as implemented in *FOX*(Favre-Nicolin & Černý, 2002 ▸) and/or *EXPO2014*(Altomare *et al.*, 2013 ▸). The entinostat mol­ecule was downloaded from PubChem (Kim *et al.*, 2023 ▸) as Conformer3D_COMPOUND_CID_4261.sdf. It was con­verted to a *.mol2 file using *Mercury* (Macrae *et al.*, 2020 ▸), and to a Fenske-Hall *Z*-matrix using *OpenBabel* (O’Boyle *et al.*, 2011 ▸). The structures were optimized using *VASP* (Kresse & Furthmüller, 1996 ▸). Since space group *Pna*2_1_ yielded the lowest energy, it was adopted for the final refinements and discussion.

Several false minima were encountered during structure solution. There were three signs that these were not the correct structure, even though *R*_wp_ was as low as 0.0466: (1) the agreement of the Rietveld-refined and DFT-optimized structure was poor (root-mean-square Cartesian displacement ∼0.9 Å – outside the normal range for correct structures); (2) the DFT optimization was very slow to converge (> 600 cycles of geometry optimization); (3) the displacement coefficients were much larger than expected (> 0.2 Å^2^). To overcome these false minima, additional cycles (183) of parallel tempering in *FOX* were carried out to yield the structure described here.

**Figure 6:**
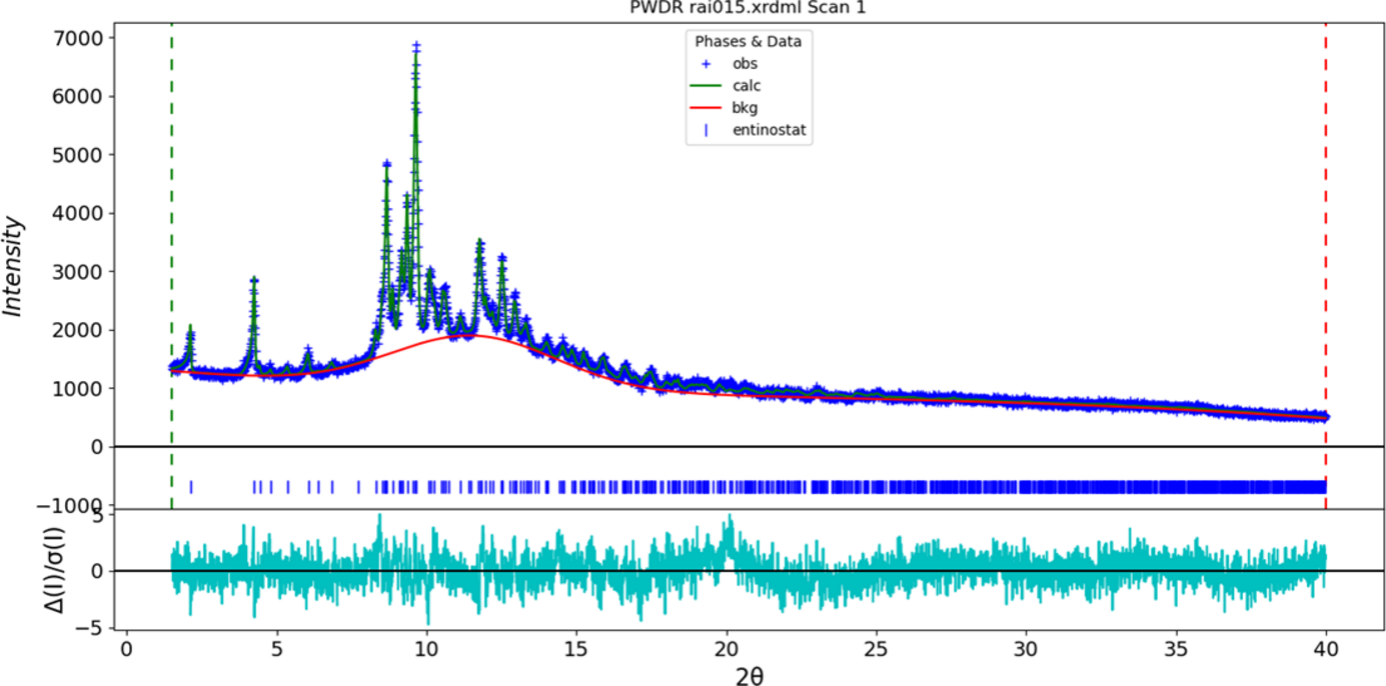
The Rietveld plot for entinostat Form B. The blue crosses represent the observed data points, and the green line is the calculated pattern. The cyan curve is the normalized error plot, and the red line is the background curve. The blue tick marks indicate the peak positions.

Rietveld refinement was carried out with *GSAS-II* (Toby & Von Dreele, 2013 ▸). Only the 1.5–40.0° portion of the pattern was included in the refinements (*d*_min_ = 1.037 Å). All non-H bond distances and angles were subjected to restraints, based on a *Mercury*/Mogul geometry check (Sykes *et al.*, 2011 ▸; Bruno *et al.*, 2004 ▸). The Mogul average and standard deviation for each qu­antity were used as the restraint parameters. The three aromatic rings were restrained to be planar. The restraints contributed 3.2% to the overall χ^2^. The hydrogen atoms were included in calculated positions, which were recalculated during the refinement using *Materials Studio* (Dassault Systèmes, 2023 ▸). The *U*_iso_ of the heavy atoms were grouped by chemical similarity. The *U*_iso_ for the H atoms were fixed at 1.3× the *U*_iso_ of the heavy atoms to which they are attached. The peak profiles were described using the generalized microstrain model (Stephens, 1999 ▸). The background was modeled using a four-term shifted Chebyshev polynomial, with a peak at 11.61° to model the scattering from the glass capillary. The final refinement of 107 variables using 4608 observations and 72 restraints yielded the residuals *R*_wp_ = 0.0697 and GOF = 1.28. The largest peak (0.19 Å from N7) and hole (1.46 Å from C10) in the difference-Fourier map are 0.61 (12) and −0.53 (12) e Å^−3^, respectively. The final Rietveld plot is shown in Fig. 6 ▸. The largest features in the normalized error plot are in the intensities of some of the peaks.

The crystal structure of entinostat was optimized (fixed experimental unit cell) with density functional techniques using *VASP* (Kresse & Furthmüller, 1996 ▸) through the *MedeA* graphical inter­face (Materials Design, 2024 ▸). The calculation was carried out on 32 cores of a 144-core (768 Gb memory) HPE Superdome Flex 280 Linux server at North Central College. The calculation used the GGA-PBE functional, a plane wave cutoff energy of 400.0 eV, and a *k*-point spacing of 0.5 Å^−1^ leading to a 1 × 2 × 3 mesh, and took ∼4.2 h. Single-point density functional calculations (fixed experimental cell) and population analysis were carried out using *CRYSTAL23* (Erba *et al.*, 2023 ▸). The basis sets for the H, C, N and O atoms in the calculation were those of Gatti *et al.* (1994 ▸). The calculations were run on a 3.5 GHz PC using 8 k-points and the B3LYP functional, and took ∼2.4 h.

## Supplementary Material

Crystal structure: contains datablock(s) Rietveld, VASP. DOI: 10.1107/S2056989025007406/hb8151sup1.cif

Supporting information file. DOI: 10.1107/S2056989025007406/hb8151Rietveldsup2.cml

CCDC references: 2481225, 2481224

Additional supporting information:  crystallographic information; 3D view; checkCIF report

## Figures and Tables

**Table 1:** Hydrogen-bond geometry (Å, °) for entinostat Form B[Chem scheme1]

*D*—H⋯*A*	*D*—H	H⋯*A*	*D*⋯*A*	*D*—H⋯*A*
N4—H35⋯N7^i^	1.05	1.86	2.906	174
N5—H36⋯O2^ii^	1.03	1.91	2.923	168
N6—H45⋯O2^ii^	1.02	2.03	3.034	166
C9—H29⋯O3^iii^	1.10	2.39	3.482	170
C25—H43⋯O3^i^	1.09	2.33	3.281	144
C26—H46⋯O1^iv^	1.09	2.34	3.199	135

Symmetry codes: (i) 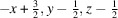; (ii) 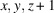; (iii) 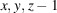; (iv) 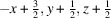.

**Table 2:** Experimental details

	Rietveld
Crystal data	
Chemical formula	C_21_H_20_N_4_O_3_
*M*_r_	376.42
Crystal system, space group	Orthorhombic, *Pna*2_1_
Temperature (K)	300
*a*, *b*, *c* (Å)	38.236 (5), 9.4459 (7), 5.0673 (4)
*V* (Å^3^)	1830.2 (2)
*Z*	4
Radiation type	Mo *K*α_1,2_, λ = 0.70932, 0.71361 Å
Specimen shape, size (mm)	Cylinder, 12 × 0.7

Data collection	
Diffractometer	PANalytical Empyrean
Specimen mounting	Glass capillary
Data collection mode	Transmission
Scan method	Step
2θ values (°)	2θ_min_ = 1.002 2θ_max_ = 49.991 2θ_step_ = 0.008

Refinement	
*R* factors and goodness of fit	*R*_p_ = 0.029, *R*_wp_ = 0.036, *R*_exp_ = 0.029, *R*(*F*^2^) = 0.11709, χ^2^ = 1.636
No. of parameters	107
No. of restraints	72
H-atom treatment	Only H-atom displacement parameters refined
(Δ/σ)_max_	0.368

Computer programs: *FOX* (Favre-Nicolin & Černý, 2002 ▸) and *GSAS-II* (Toby & Von Dreele, 2013 ▸).

## supplementary crystallographic information

### Pyridin-3-ylmethyl N-({4-[(2-aminophenyl)carbamoyl]phenyl}methyl)carbamate (Rietveld) Crystal data

**Table:**

C_21_H_20_N_4_O_3_	*V* = 1830.2 (2) Å^3^
*M**_r_* = 376.42	*Z* = 4
Orthorhombic, *P**n**a*2_1_	*D*_x_ = 1.366 Mg m^−^^3^
*a* = 38.236 (5) Å	Mo *K*α_1,2_ radiation, λ = 0.70932, 0.71361 Å
*b* = 9.4459 (7) Å	*T* = 300 K
*c* = 5.0673 (4) Å	cylinder, 12 × 0.7 mm

### Pyridin-3-ylmethyl N-({4-[(2-aminophenyl)carbamoyl]phenyl}methyl)carbamate (Rietveld) Data collection

**Table:**

PANalytical Empyrean diffractometer	Data collection mode: transmission
Radiation source: sealed X-ray tube	Scan method: step
Zr filter monochromator	2θ_min_ = 1.002°, 2θ_max_ = 49.991°, 2θ_step_ = 0.008°
Specimen mounting: glass capillary	

### Pyridin-3-ylmethyl N-({4-[(2-aminophenyl)carbamoyl]phenyl}methyl)carbamate (Rietveld) Refinement

**Table:**

Least-squares matrix: full	72 restraints
*R*_p_ = 0.029	23 constraints
*R*_wp_ = 0.036	Only H-atom displacement parameters refined
*R*_exp_ = 0.029	Weighting scheme based on measured s.u.'s
*R*(*F*^2^) = 0.11709	(Δ/σ)_max_ = 0.368
5864 data points	Background function: Background function: "chebyschev-1" function with 4 terms: 871.1(20), -360.1(18), 11.3(15), -45.5(16), Background peak parameters: pos, int, sig, gam: 11.611(13), 6.24(5)e5, 7.58(9)e4, 0.100,
Profile function: Finger-Cox-Jephcoat function parameters U, V, W, X, Y, SH/L: peak variance(Gauss) = Utan(Th)^2^+Vtan(Th)+W: peak HW(Lorentz) = X/cos(Th)+Ytan(Th); SH/L = S/L+H/L U, V, W in (centideg)^2^, X & Y in centideg 25.531, 9.905, 0.000, 1.091, 3.951, 0.034,	Preferred orientation correction: Simple spherical harmonic correction Order = 2 Coefficients: 0:0:C(2,0) = 0.103(29); 0:0:C(2,2) = -0.018(21)
107 parameters	

### Pyridin-3-ylmethyl N-({4-[(2-aminophenyl)carbamoyl]phenyl}methyl)carbamate (Rietveld) Fractional atomic coordinates and isotropic or equivalent isotropic displacement parameters (Å^2^)

**Table:**

	*x*	*y*	*z*	*U*_iso_*/*U*_eq_	
O1	0.7170 (5)	0.3108 (13)	1.26983	0.055 (8)*	
O2	0.5094 (5)	0.313 (3)	0.331 (4)	0.029 (9)*	
O3	0.7017 (6)	0.5418 (13)	1.292 (8)	0.055 (8)*	
N4	0.6773 (5)	0.393 (2)	0.994 (5)	0.055 (8)*	
N5	0.4935 (5)	0.278 (3)	0.760 (5)	0.029 (9)*	
N6	0.4756 (7)	0.052 (3)	1.089 (7)	0.055 (10)*	
N7	0.8163 (6)	0.5927 (17)	1.337 (8)	0.033 (9)*	
C8	0.6206 (3)	0.4434 (17)	0.780 (5)	0.027 (8)*	
C9	0.6556 (6)	0.500 (2)	0.869 (8)	0.055 (8)*	
C10	0.5512 (3)	0.3708 (19)	0.659 (5)	0.027 (8)*	
C11	0.6038 (4)	0.502 (3)	0.563 (5)	0.027 (8)*	
C12	0.6023 (5)	0.347 (4)	0.933 (6)	0.027 (8)*	
C13	0.5696 (3)	0.467 (2)	0.504 (5)	0.027 (8)*	
C14	0.5681 (5)	0.311 (3)	0.875 (6)	0.027 (8)*	
C15	0.5163 (4)	0.318 (3)	0.568 (4)	0.029 (9)*	
C16	0.4613 (4)	0.207 (2)	0.725 (6)	0.055 (10)*	
C17	0.4533 (7)	0.091 (3)	0.889 (8)	0.055 (10)*	
C18	0.4383 (9)	0.246 (4)	0.523 (9)	0.055 (10)*	
C19	0.6981 (5)	0.4247 (11)	1.198 (5)	0.055 (8)*	
C20	0.7430 (4)	0.335 (2)	1.474 (4)	0.055 (8)*	
C21	0.4222 (8)	0.017 (3)	0.844 (9)	0.055 (10)*	
C22	0.7750 (5)	0.4006 (19)	1.352 (8)	0.033 (9)*	
C23	0.4077 (7)	0.169 (4)	0.483 (8)	0.055 (10)*	
C24	0.3997 (8)	0.056 (4)	0.643 (8)	0.055 (10)*	
C25	0.7929 (8)	0.335 (3)	1.149 (7)	0.033 (9)*	
C26	0.7880 (8)	0.529 (2)	1.439 (7)	0.033 (9)*	
C27	0.8221 (8)	0.398 (3)	1.040 (9)	0.033 (9)*	
C28	0.8329 (6)	0.526 (2)	1.140 (7)	0.033 (9)*	
H29	0.67008	0.54448	0.69050	0.0713*	
H30	0.65046	0.58991	1.01441	0.0713*	
H31	0.62060	0.54691	0.39817	0.0356*	
H32	0.61567	0.29902	1.10662	0.0356*	
H33	0.55888	0.51030	0.32747	0.0356*	
H34	0.55238	0.26635	1.04151	0.0356*	
H35	0.67159	0.28737	0.92867	0.0713*	
H36	0.50674	0.20423	0.89818	0.0372*	
H37	0.45017	0.32241	0.38325	0.0721*	
H38	0.74850	0.23110	1.55041	0.0713*	
H39	0.73009	0.39869	1.62195	0.0713*	
H40	0.41682	−0.08211	0.94296	0.0721*	
H41	0.39034	0.23483	0.36479	0.0721*	
H42	0.37377	0.02234	0.63785	0.0721*	
H43	0.78356	0.22806	1.07112	0.0432*	
H44	0.47309	−0.06399	1.12476	0.0721*	
H45	0.46876	0.11006	1.27176	0.0721*	
H46	0.77066	0.58946	1.57984	0.0432*	
H47	0.83386	0.33515	0.89816	0.0432*	
H48	0.85627	0.56225	1.10069	0.0432*	

### Pyridin-3-ylmethyl N-({4-[(2-aminophenyl)carbamoyl]phenyl}methyl)carbamate (Rietveld) Geometric parameters (Å, º)

**Table:**

O1—C19	1.345 (3)	C19—O3	1.213 (2)
O1—C20	1.453 (3)	C19—N4	1.337 (3)
O2—C15	1.229 (3)	C20—O1	1.453 (3)
O3—C19	1.213 (2)	C20—C22	1.503 (3)
N4—C9	1.454 (2)	C20—H38	1.076 (19)
N4—C19	1.337 (3)	C20—H39	1.08 (2)
N4—H35	1.08 (2)	C21—C17	1.399 (3)
N5—C15	1.361 (4)	C21—C24	1.383 (2)
N5—C16	1.416 (3)	C21—H40	1.081 (19)
N5—H36	1.11 (3)	C22—C20	1.503 (3)
N6—C17	1.376 (4)	C22—C25	1.382 (3)
N6—H44	1.11 (2)	C22—C26	1.382 (3)
N6—H45	1.11 (4)	C23—C18	1.387 (2)
N7—C26	1.342 (3)	C23—C24	1.376 (3)
N7—C28	1.340 (3)	C23—H41	1.09 (3)
C8—C9	1.511 (3)	C24—C21	1.383 (2)
C8—C11	1.386 (2)	C24—C23	1.376 (3)
C8—C12	1.386 (2)	C24—H42	1.042 (19)
C9—N4	1.454 (2)	C25—C22	1.382 (3)
C9—C8	1.511 (3)	C25—C27	1.382 (2)
C9—H29	1.14 (4)	C25—H43	1.140 (15)
C9—H30	1.14 (4)	C26—N7	1.342 (3)
C10—C13	1.389 (2)	C26—C22	1.382 (3)
C10—C14	1.390 (2)	C26—H46	1.13 (3)
C10—C15	1.498 (3)	C27—C25	1.382 (2)
C11—C8	1.386 (2)	C27—C28	1.373 (3)
C11—C13	1.384 (2)	C27—H47	1.04 (3)
C11—H31	1.14 (2)	C28—N7	1.340 (3)
C12—C8	1.386 (2)	C28—C27	1.373 (3)
C12—C14	1.385 (2)	C28—H48	0.977 (17)
C12—H32	1.110 (17)	H29—C9	1.14 (4)
C13—C10	1.389 (2)	H30—C9	1.14 (4)
C13—C11	1.384 (2)	H31—C11	1.14 (2)
C13—H33	1.065 (18)	H32—C12	1.110 (17)
C14—C10	1.390 (2)	H33—C13	1.065 (18)
C14—C12	1.385 (2)	H34—C14	1.12 (2)
C14—H34	1.12 (2)	H35—N4	1.08 (2)
C15—O2	1.229 (3)	H36—N5	1.11 (3)
C15—N5	1.361 (4)	H37—C18	1.11 (3)
C15—C10	1.498 (3)	H38—C20	1.076 (19)
C16—N5	1.416 (3)	H39—C20	1.08 (2)
C16—C17	1.4050 (16)	H40—C21	1.081 (19)
C16—C18	1.395 (2)	H41—C23	1.09 (3)
C17—N6	1.376 (4)	H42—C24	1.042 (19)
C17—C16	1.4050 (16)	H43—C25	1.140 (15)
C17—C21	1.399 (3)	H44—N6	1.11 (2)
C18—C16	1.395 (2)	H45—N6	1.11 (4)
C18—C23	1.387 (2)	H46—C26	1.13 (3)
C18—H37	1.11 (3)	H47—C27	1.04 (3)
C19—O1	1.345 (3)	H48—C28	0.977 (17)
			
C19—O1—C20	115.7 (3)	C17—C16—C18	120.10 (11)
C9—N4—C19	121.6 (3)	N6—C17—C16	120.91 (14)
C9—N4—H35	113.2 (7)	N6—C17—C21	120.61 (15)
C19—N4—H35	124.4 (12)	C16—C17—C21	118.48 (10)
C15—N5—C16	126.8 (3)	C16—C18—C23	119.96 (16)
C15—N5—H36	109.5 (16)	C16—C18—H37	112.5 (17)
C16—N5—H36	100.2 (10)	C23—C18—H37	126.2 (18)
C17—N6—H44	109 (4)	O1—C19—O3	124.2 (2)
C17—N6—H45	109 (3)	O1—C19—N4	110.5 (2)
H44—N6—H45	109 (3)	O3—C19—N4	125.00 (19)
C26—N7—C28	117.34 (18)	O1—C20—C22	109.4 (3)
C9—C8—C11	120.4 (3)	O1—C20—H38	104.3 (11)
C9—C8—C12	120.7 (3)	C22—C20—H38	111.5 (18)
C11—C8—C12	118.22 (13)	O1—C20—H39	105.5 (9)
N4—C9—C8	113.0 (3)	C22—C20—H39	115.3 (14)
N4—C9—H29	109 (3)	H38—C20—H39	110.2 (17)
C8—C9—H29	108.9 (15)	C17—C21—C24	120.91 (13)
N4—C9—H30	109.5 (15)	C17—C21—H40	121 (2)
C8—C9—H30	108 (3)	C24—C21—H40	117 (3)
H29—C9—H30	108.9 (14)	C20—C22—C25	121.4 (2)
C13—C10—C14	118.34 (12)	C20—C22—C26	121.5 (2)
C13—C10—C15	119.6 (3)	C25—C22—C26	117.08 (14)
C14—C10—C15	121.4 (3)	C18—C23—C24	120.38 (13)
C8—C11—C13	121.06 (13)	C18—C23—H41	108 (2)
C8—C11—H31	118.0 (13)	C24—C23—H41	129 (3)
C13—C11—H31	117.6 (12)	C21—C24—C23	120.16 (12)
C8—C12—C14	120.99 (13)	C21—C24—H42	122 (3)
C8—C12—H32	118.7 (11)	C23—C24—H42	116 (3)
C14—C12—H32	120.3 (11)	C22—C25—C27	120.07 (15)
C10—C13—C11	120.70 (12)	C22—C25—H43	120.0 (11)
C10—C13—H33	122.3 (9)	C27—C25—H43	119.8 (13)
C11—C13—H33	116.9 (9)	N7—C26—C22	123.98 (18)
C10—C14—C12	120.69 (12)	N7—C26—H46	119.3 (15)
C10—C14—H34	119.9 (13)	C22—C26—H46	115.8 (14)
C12—C14—H34	115.9 (18)	C25—C27—C28	118.54 (16)
O2—C15—N5	123.4 (3)	C25—C27—H47	112.3 (15)
O2—C15—C10	120.3 (2)	C28—C27—H47	129.0 (15)
N5—C15—C10	116.3 (2)	N7—C28—C27	123.0 (2)
N5—C16—C17	118.9 (2)	N7—C28—H48	115 (2)
N5—C16—C18	121.0 (2)	C27—C28—H48	121 (2)

### (VASP) Crystal data

**Table:**

C21H20N4O_3_	*b* = 9.44590 Å
*M**_r_* = 376.42	*c* = 5.06730 Å
Orthorhombic, *P**n**a*2_1_	*V* = 1830.17 Å^3^
*a* = 38.23600 Å	*Z* = 4

### (VASP) Data collection

**Table:**

DFT optimization	*k* = →
*h* = →	*l* = →

### (VASP) Fractional atomic coordinates and isotropic or equivalent isotropic displacement parameters (Å^2^)

**Table:**

	*x*	*y*	*z*	*B*_iso_*/*B*_eq_	
O1	0.71602	0.30908	1.29383		
O2	0.50809	0.30967	0.33129		
O3	0.70419	0.54791	1.27319		
N4	0.67730	0.38675	0.99681		
N5	0.49333	0.28080	0.76790		
N6	0.47635	0.04721	1.08292		
N7	0.81620	0.59379	1.32902		
C8	0.62069	0.44093	0.78248		
C9	0.65707	0.49049	0.85136		
C10	0.55157	0.36158	0.65553		
C11	0.60342	0.50113	0.56549		
C12	0.60305	0.33908	0.93232		
C13	0.56950	0.46114	0.50143		
C14	0.56879	0.30057	0.87182		
C15	0.51586	0.31652	0.57107		
C16	0.46077	0.21116	0.73033		
C17	0.45309	0.09247	0.89166		
C18	0.43725	0.25439	0.53534		
C19	0.69932	0.42603	1.19110		
C20	0.74344	0.33754	1.48371		
C21	0.42225	0.01638	0.83831		
C22	0.77550	0.39878	1.35610		
C23	0.40654	0.17878	0.48894		
C24	0.39948	0.05794	0.63863		
C25	0.79359	0.32785	1.15597		
C26	0.78808	0.53075	1.43545		
C27	0.82290	0.39187	1.04445		
C28	0.83313	0.52485	1.13556		
H29	0.67074	0.52182	0.66856		
H30	0.65555	0.58672	0.97420		
H31	0.61678	0.57992	0.44388		
H32	0.61618	0.29023	1.10028		
H33	0.55649	0.50636	0.33008		
H34	0.55578	0.22174	0.99486		
H35	0.67774	0.28113	0.93441		
H36	0.50169	0.29696	0.95825		
H37	0.44352	0.34670	0.41575		
H38	0.74910	0.23349	1.57088		
H39	0.73357	0.40837	1.63868		
H40	0.41672	−0.07745	0.95741		
H41	0.38877	0.21199	0.33265		
H42	0.37603	−0.00446	0.59901		
H43	0.78509	0.22433	1.08477		
H44	0.46617	−0.02342	1.21369		
H45	0.48953	0.12451	1.18386		
H46	0.77497	0.58984	1.59210		
H47	0.83767	0.33956	0.88829		
H48	0.85582	0.57876	1.05182		

### (VASP) Hydrogen-bond geometry (Å, º)

**Table:**

*D*—H···*A*	*D*—H	H···*A*	*D*···*A*	*D*—H···*A*
N4—H35···N7^i^	1.05	1.86	2.906	174
N5—H36···O2^ii^	1.03	1.91	2.923	168
N6—H45···O2^ii^	1.02	2.03	3.034	166
C9—H29···O3^iii^	1.10	2.39	3.482	170
C25—H43···O3^i^	1.09	2.33	3.281	144
C26—H46···O1^iv^	1.09	2.34	3.199	135

Symmetry codes: (i) −*x*+3/2, *y*−1/2, *z*−1/2; (ii) *x*, *y*, *z*+1; (iii) *x*, *y*, *z*−1; (iv) −*x*+3/2, *y*+1/2, *z*+1/2.
